# Carbonic Anhydrases: An Ancient Tool in Calcareous Sponge Biomineralization

**DOI:** 10.3389/fgene.2021.624533

**Published:** 2021-04-07

**Authors:** Oliver Voigt, Benedetta Fradusco, Carolin Gut, Charalampos Kevrekidis, Sergio Vargas, Gert Wörheide

**Affiliations:** ^1^Department of Earth and Environmental Sciences, Palaeontology and Geobiology, Ludwig-Maximilians-Universität München, Munich, Germany; ^2^GeoBio-Center, Ludwig-Maximilians-Universität München, Munich, Germany; ^3^SNSB-Bayerische Staatssammlung für Paläontologie und Geologie, Munich, Germany

**Keywords:** carbonic anhydrases, Porifera: Calcarea, biomineralization and calcification, evolution, spicule formation

## Abstract

Enzymes of the α-carbonic anhydrase gene family (CAs) are essential for the deposition of calcium carbonate biominerals. In calcareous sponges (phylum Porifera, class Calcarea), specific CAs are involved in the formation of calcite spicules, a unique trait and synapomorphy of this class. However, detailed studies on the CA repertoire of calcareous sponges exist for only two species of one of the two Calcarea subclasses, the Calcaronea. The CA repertoire of the second subclass, the Calcinea, has not been investigated so far, leaving a considerable gap in our knowledge about this gene family in Calcarea. Here, using transcriptomic analysis, phylogenetics, and *in situ* hybridization, we study the CA repertoire of four additional species of calcareous sponges, including three from the previously unsampled subclass Calcinea. Our data indicate that the last common ancestor of Calcarea had four ancestral CAs with defined subcellular localizations and functions (mitochondrial/cytosolic, membrane-bound, and secreted non-catalytic). The evolution of membrane-bound and secreted CAs involved gene duplications and losses, whereas mitochondrial/cytosolic and non-catalytic CAs are evidently orthologous genes. Mitochondrial/cytosolic CAs are biomineralization-specific genes recruited for biomineralization in the last common ancestor of calcareous sponges. The spatial–temporal expression of these CAs differs between species, which may reflect differences between subclasses or be related to the secondary thickening of spicules during biomineralization that does not occur in all species. With this study, we extend the understanding of the role and the evolution of a key biomineralization gene in calcareous sponges.

## Introduction

Animal biomineralization is a controlled process and leads to the production of mineral–organic composite materials that considerably differ in shape and material properties from their purely inorganic counterparts. The ability to form functional biominerals, such as endo- and exoskeletons, protective shells, or teeth, had been a significant step in animal evolution. Calcium carbonate biomineralization, the most widespread type among animal phyla (Murdock and Donoghue, 2011), evolved several times independently, resulting in multiple recruitments of the same genes for biomineralization in different lineages (Murdock, 2020). Among these genes, members of the α-carbonic anhydrase gene family (CAs) are essential for biomineralization (Le Roy et al., 2014). CAs are zinc-binding enzymes that catalyze the reversible conversion of carbon dioxide and water to bicarbonate and one proton (Tripp et al., 2001). The zinc-binding is mediated by three histidine residues essential for the protein’s catalytic function (Aspatwar et al., 2014; Kim et al., 2020). CAs are involved in many physiological processes requiring ion regulation or carbon transport (Supuran, 2016), both of which are crucial for the controlled precipitation of carbonate biominerals. In mammals, where they are best studied, 16 different CAs are expressed in specific tissues and active in defined subcellular compartments (Imtaiyaz Hassan et al., 2013). Cytosolic, mitochondrial, membrane-bound, and secreted CA forms can be distinguished, and these groups got expanded and reduced in different animal groups (Le Roy et al., 2014; Voigt et al., 2014). Specific CAs are involved in the carbonate biomineralization in distinct metazoan lineages (reviewed in Le Roy et al., 2014), including sponges (Jackson et al., 2007; Voigt et al., 2014; Germer et al., 2015).

Among extant sponges, only the calcareous sponges (class Calcarea) can produce calcite spicules, whereas other classes’ spicules are siliceous. Some lineages among demosponges and a few calcareans have massive calcium carbonate basal skeletons, the so-called coralline sponges or sclerosponges. The biomineralizing CAs used by carbonate-producing demosponges are not orthologous to the CAs involved in the spicule formation of calcareous sponges (Voigt et al., 2014), suggesting that the two biomineralization types evolved independently. This observation agrees with the idea that the formation of calcitic spicules is an evolutionary innovation of calcareous sponges (Manuel, 2006).

**FIGURE 1:**
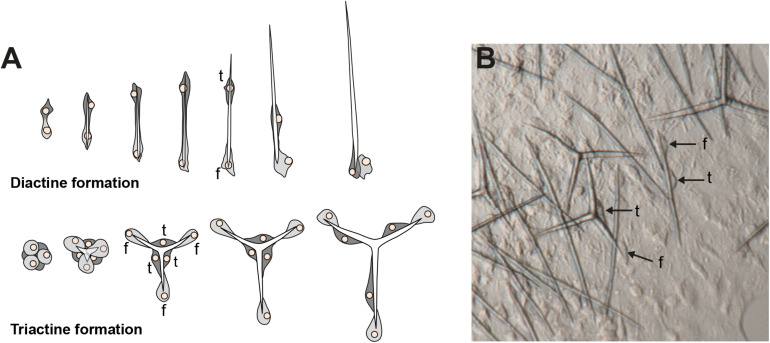
Spicule formation by sclerocytes in calcareous sponges; **(A)** Movement of founder cell (*f*) and thickener (*t*) cells during diactine and triactine formation; **(B)** *in vivo* formation of spicules by sclerocytes (*f* = founder cell, *t* = thickener cell). Modified from Voigt et al. (2017).

The shapes of calcareous sponge spicules are simple compared with the sometimes very elaborate siliceous spicules found in the other sponge classes. With only a few exceptions, calcareous sponge spicules can be of three basic types: monaxonic, two-tipped diactines, triactines with three spicules rays, and four-rayed tetractines. Specialized cells, the sclerocytes, produce these spicules, and only a few sclerocytes interact in the formation of one specific spicule: Two sclerocytes produce a diactine, six sclerocytes form a triactine, and seven a tetractines (Minchin, 1898; Woodland, 1905; Ledger and Jones, 1977). A pair of sclerocytes is involved in the growth of each actine of these spicules. After an initial phase, the so-called founder cell promotes actine elongation, the second, so-called thickener cell in some, but not all species deposit additional calcium carbonate on the actine, as it migrates back toward the founder cell (Figure 1, Ledger and Jones, 1977; Ilan et al., 1996). Calcareous sponges can possess only one or any combination of the three spicule types in their body, and in many cases, certain spicule types are restricted to specific body parts. This indicates that spicule formation is under strict genetic control in calcareous sponges, and specific CAs play an essential role in this genetic control (Voigt et al., 2017). Indeed, biomineralizing CAs were identified in Calcaronea, one of the two subclasses of calcareous sponges (Voigt et al., 2014). In each of the two studied species, *Sycon ciliatum* (Sci) and *Leucosolenia complicata* (Lco), sclerocytes express one intracellular CA (SciCA1 and LcoCA1) and one secreted or membrane-bound CA (SciCA2 and LcoCA3) during spicule formation. In *Sycon*, these two CAs have specific spatial and temporal expression patterns during spicule formation: Although early in spicule formation, all sclerocytes express *SciCA1* and *SciCA2*, in later stages, only SciCA2 is produced in the founder cells. Simultaneously, the production of certain spicular matrix proteins is induced in the thickener cells, indicating an orchestrated regulation of biomineralization gene expression during spicule formation (Voigt et al., 2017). In addition to these two sclerocyte-specific CAs, several additional secreted or membrane-bound CA proteins are present in both species (six in *Sycon* and four in *Leucosolenia*) and are not directly involved in the biomineralization process (Voigt et al., 2014). Some of these probably lost their catalytic activity due to substitutions of the zinc-binding histidine residues. Such inactive proteins of the gene family are called carbonic anhydrase-related proteins (CARPs, Aspatwar et al., 2014). Determination of gene orthology is difficult for the secreted CAs because of the several gene duplications and losses during evolution that shaped this gene family (Voigt et al., 2014). Phylogenetic analysis of the CAs from the subclass Calcaronea implied the presence of at least three ancestral CAs in the last common ancestor of this subclass (Voigt et al., 2014). Conclusions about the set of CAs in the last common ancestor of all extant calcareous sponges, however, require the study of additional species from the second calcarean subclass, the Calcinea. To gain further insights into the evolution of these essential biomineralization genes of calcareous sponges, we explored the CA repertoire of four additional species from both subclasses by transcriptomic, phylogenetics, and *in situ* hybridization (ISH) experiments.

## Methods

### Sampling, RNA Extraction, and Transcriptome Sequencing

RNA of two species of the subclass Calcinea was extracted. The first species was isolated from our laboratory aquarium system and belonged to the genus *Clathrina sensu lato*. The genus *Clathrina* was recently revised (Klautau et al., 2013), but the species belongs to a yet unnamed clade of calcareous sponges that, in contrast to the new definition of the genus, bears tetractines in addition to triactines. Therefore, in this work, we refer to it as *Clathrina* sp. (Csp) in the sense of *Clathrina sensu lato*. It is an asconoid sponge whose body consists of thin anastomosed tubes. Small living specimens were incubated for 18 h in calcein in seawater to stain spicules produced in this time frame and confirm ongoing biomineralization as described before (Voigt et al., 2014). Other specimens were processed for RNA extraction or fixed for RNA ISH, according to previously described methods (Fortunato et al., 2012).

The second calcinean species was *Pericharax orientalis* (Por), sampled at the MaRHE center in the Fafuu Atoll in the Maldives. RNA of both species was isolated using Trizol. RNA quality was verified with an Agilent Bioanalyzer 2,100, and transcriptomic libraries were prepared with the Illumina TruSeq2 kit (*Clathrina* s. l.) or the Lexogen SENSE Total RNA-Seq Library Prep Kit. Sequencing was performed on an Illumina Miniseq, NextSeq, and 1,500 HiSeq Sequencer.

### Assembly and Identification of α-Carbonic Anhydrases

In addition to the newly sequenced species, published raw reads of two species from a phylogenomic study (Simion et al., 2017), *Clathrina coriacea* (Cco and subclass Calcinea) and *Grantia compressa* (Gco subclass Calcaronea), were downloaded from the GenBank short read archive (SRX1719631 and SRX1719634, respectively). The obtained raw reads were quality controlled, trimmed, and assembled with Trinity (Grabherr et al., 2011); ORFs were predicted for the Trinity contigs with TransDecoder v.5.0.0^1^ and used to create a Blast database in Geneious Prime 2019^2^. Raw reads of transcriptomes were submitted to ENA short read archive (Study accession PRJEB41034). Assemblies of transcriptomes are available at LMU Open Data ( 10.5282/ubm/data.202).

Protein sequences of *S. ciliatum* CA1 and CA9 (SciCA1, SciCA2, Voigt et al., 2014) were used as BLAST queries against these libraries. Only hits that were confirmed to be CAs by blasting them against Swiss Prot (Katoh and Standley, 2013) were considered further. Of these, we manually corrected some 5′ partial Transdecoder predictions because the potential CDS was close to the 5’ end of the contig and comparison with other CAs suggested (see alignments) that the start ATG was included and that the coding sequence was indeed complete (Supplementary Table 1). As a measurement of each CA’s expression level, their fragments per kilobase million (FPKM) values were obtained with RSEM using Bowtie2 (Li and Dewey, 2011) in a Galaxy environment (Afgan et al., 2018) by mapping the reads back to the obtained transcriptome assemblies. We used SignalP 5.0 (Armenteros et al., 2019) to identify the presence of signal peptides, hence whether a protein is secreted or not. TargetP (Emanuelsson et al., 2000) was used to predict the subcellular localization of calcareous sponge CAs. The presence of a glycosylphosphatidylinositol (GPI) anchor, indicative for membrane-bound CAs, was determined with PredGPI (Pierleoni et al., 2008).

### Phylogenetic Analysis

We complemented the dataset of identified CA amino acid sequences with published calcareous sponge CAs from *S. ciliatum* and *L. complicata* and CAs from other sponge classes (Supplementary Table 2) and from selected metazoans with sequenced genomes (*Homo sapiens*, *Strongylocentrotus purpurea*, and *Mnemiopsis leidyi*), and the scleractinian coral *Stylophora pistillata*. Non-metazoan CAs (from the green algae *Chlamydomonas* and two Enterobacteria) were added as an outgroup. Sequences were aligned with MAFFT (G-INS-i, gap-opening penalty 3, Katoh and Standley, 2013). We considered one partial CA of each of the transcriptomes of *P. orientalis* and *C. coriacea* to originate from commensals because they did not group with other CAs of calcareous sponges and were only partial transcripts with low FPKM values (Supplementary Table 1). We excluded them from further analysis and also some variants of other CAs with FPKM of 0. Gblocks (Castresana, 2000) was used to select 205 sites for the phylogenetic analyses. The final alignment, including the information of the selected sites, is available from LMU Open Data ( 10.5282/ubm/data.202) as a mase-file and can be inspected with the Seaview alignment editor (Gouy et al., 2010). A maximum-likelihood tree was calculated with PhyML v3.0 (Guindon et al., 2010) using the best fitting model (LG + G) determined using the AIC in ProTest (Darriba et al., 2011). A Bayesian phylogeny was calculated with MrBayes (Ronquist and Huelsenbeck, 2003) using the same model, two runs and four chains each of five million generations. The temperature setting for the heated chains was decreased from the default of 0.1 to 0.05 to obtain better mixing. Every 1,000th tree was sampled, and a consensus tree was calculated with the sumt command with the first 25% of trees discarded as burn-in. CAs of calcareous sponges that were from the same Trinity sequence cluster but assembled as different “genes” or “isoforms” were considered to be one “gene” and collapsed in the phylogeny as they formed one clade with only short internal branches (Supplementary Table 1). Although some of these variants may be true isoforms or real genes, at least some appear to be assembly artifacts because several coded for incomplete proteins or had low FPKM values (Supplementary Table 1).

### Amplification of α-Carbonic Anhydrases and Preparation of RNA Probes, RNA in situ Hybridization

DNA and RNA were isolated from another specimen of *Clathrina* s. l. using the ZR-Duet^TM^ DNA/RNA MiniPrep (Zymo Research). Complementary DNA was generated using the extracted RNA and the ProtoScript(R) II First-Strand Complementary DNA Synthesis Kit (NEB) and used as a template in PCRs with gene-specific primers to amplify all six *Clathrina* sp. CAs (Supplementary Table 1). PCR products were cloned into the pCR4-TOPO vector (Invitrogen) and sequenced to determine the insert orientation (presence of T3 or T7 initiation site on the 3’ end of the gene’s sense strand). An additional PCR with the corresponding reverse vector primer and a probe-specific forward primer provided the template for the synthesis of DIG-labeled RNA probes (DIG RNA Labeling Mix, Roche) with the corresponding RNA polymerase to generate antisense probes (T3 or T7 polymerase, Promega). RNA ISH was performed as previously described (Fortunato et al., 2014) on fixed tissues of complete small specimens of *Clathrina* sp. The expression patterns of the different CAs were documented using a Leica FM16 stereomicroscope and a Leica DMLB compound microscope. To increase the depth of field, stacks of images were combined with the Auto-Blend-Layers function of Adobe Photoshop 2020.

## Results

**FIGURE 2:**
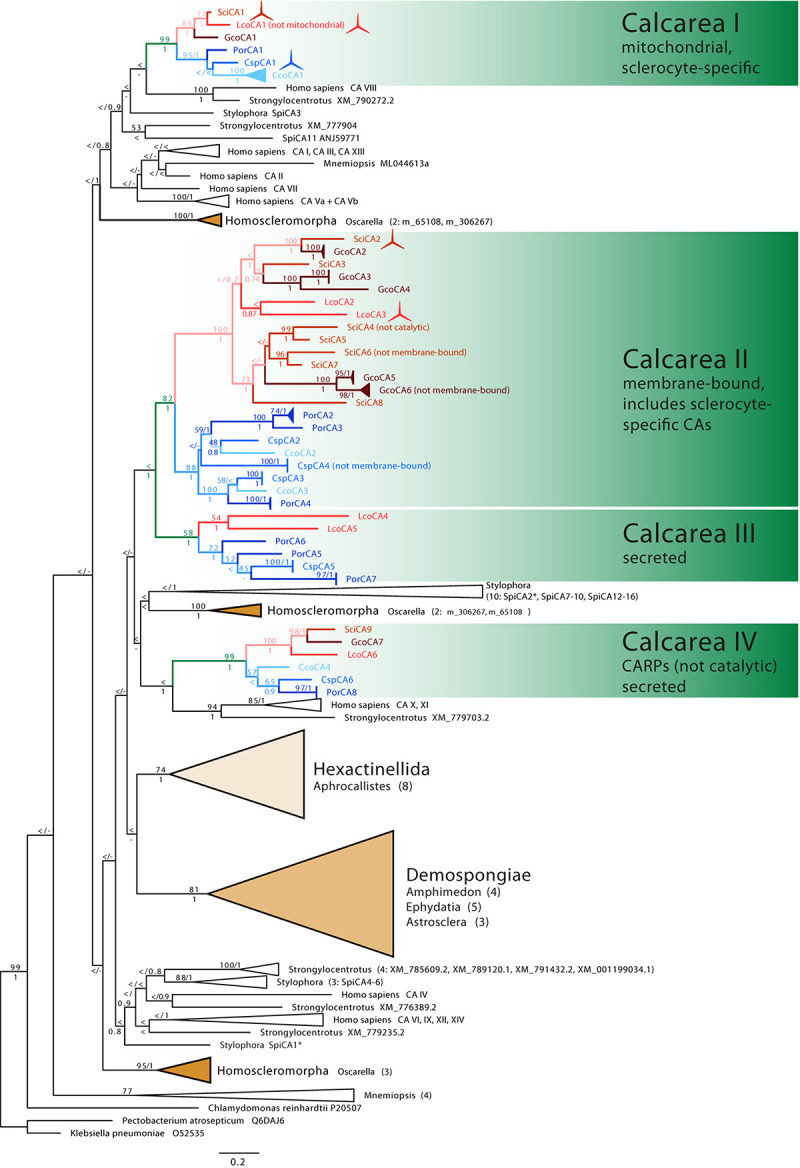
Phylogenetic tree (ML) of CAs. Sponge CAs are highlighted in color: Class Calcarea: green, subclass Calcaronea: red, subclass Calcinea: blue, remaining sponge classes shown as triangles representing their diversity. Monophyletic clades of other species’ CAs are collapsed (number of CAs is provided in brackets). ML bootstrap (*n* = 200) >50 and posterior probability >0.5 are displayed at the corresponding nodes. Spicule symbols mark CAs with verified sclerocyte-specific expression.

In the assembled transcriptomes, we identified several complete and incomplete CA genes (in a sense described in M&Ms): Six in *Clathrina* sp. (CspCA1-CspCA6), eight in *P. orientalis* (PorCA1-PorCA8), four in *C. coriacea* (CcoCA1-CcoCA4), and seven in *G. compressa* (GcoCA1–GcoCA7). We arbitrarily labeled them regarding their position in the phylogenetic tree (Figure 2), and except for CA1, the numbers do not reflect orthology among the species.

**FIGURE 3:**
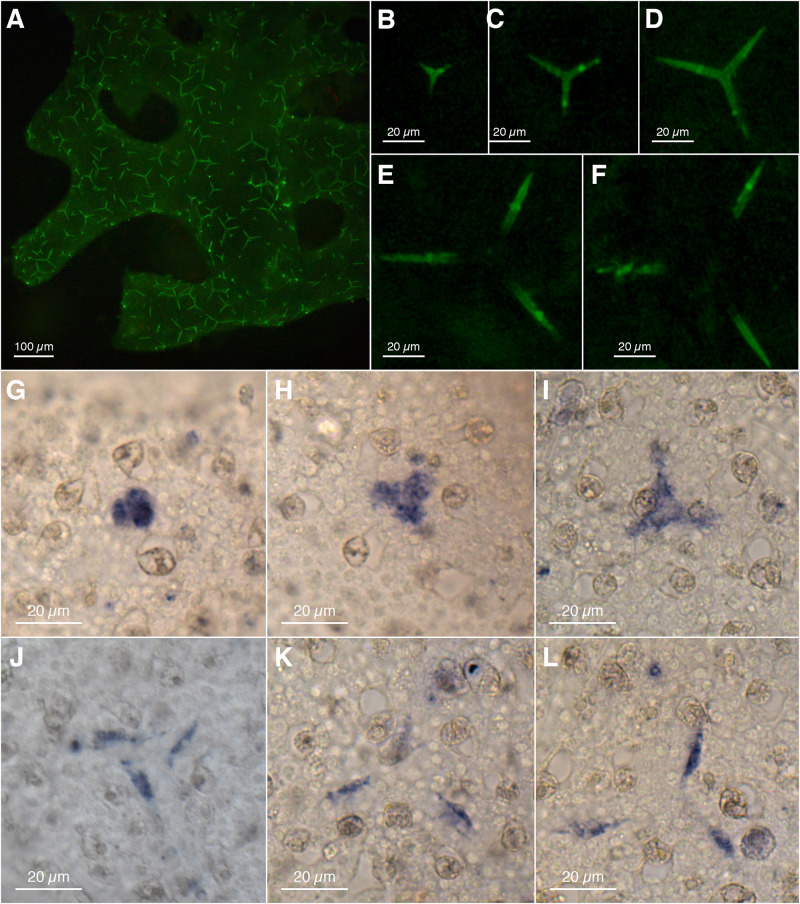
Spicule formation and CA1 expression in *Clathrina* sp.: **(A–F)** spicules formed within 18 h (calcein labeling, fluorescence microscopy); **(A)** Overview, **(B–F)** progressing stages of spicule formation, isolated from **(A)** (overlapping fluorescent actines from adjacent spicules retouched for clarity). **(G–L)** Expression of CA1 in sclerocytes with progressing spicule formation (spicules dissolved during ISH). Sclerocytes in **(J–L)** are thickener cells on the site of the spicules’ actines.

Our ISH experiments with *Clathrina* sp. revealed a sclerocyte-specific expression of *CspCA1* (Figure 3) that matches the distribution of active spicule formation expected from the calcein-staining experiments (Figure 3A and Supplementary Figure 3). Also, different spicule formation stages are recognizable by calcein-staining (Figures 3B–F) and the detailed *CspCA1* expression patterns (Figures 3G–L).

CspCA1 is not only expressed during early spicule formation stages (Figures 3G–H) but additionally in thickener cells during the later stages (Figures 3J–L). No sclerocyte-specific expression patterns were observed for *CspCA2*, *CspCA3*, and *CspCA5* (Supplementary Figure 3). For *CspCA4* and *CspCA6*, we did not detect a signal in the RNA ISH experiments (Supplementary Figure 3). These two CAs showed the lowest expression levels among the CAs of this species (Supplementary Table 1).

In the CA phylogeny, many deeper nodes have only low support values (Figure 2). The relationship of coral CAs of *S. pistillata* (SpiCA1–16, Figure 2) to calcareous sponge CAs, therefore, remains unclear, but they are not specifically closely related. Sponge CAs are not monophyletic. The CAs of the sponge classes Demospongiae and Hexactinellida are each monophyletic. They are sister clades in the ML analysis (with low bootstrap support), but their relationships to each other remain unresolved in the Bayesian reconstruction. CAs of the sponge class Homoscleromorpha occur in three distinct clades. CAs of calcareous sponges fall into four clades (Calcarea clades I–IV, Figure 2). Each of these contains CAs with predominantly the same subcellular localization and is subdivided into monophyletic clades of calcinean and calcaronean CAs.

Clade I comprises catalytic CAs (Supplementary Table 1) without signal peptide (Supplementary Table 2) and, except for LcoCA1, with a mitochondrial targeting sequence. A single CA of each species is present. In addition to CspCA1 (see discussion earlier), a sclerocyte-specific expression is documented for SciCA1 and LcoCA1 (Voigt et al., 2014), suggesting a direct involvement of clade I CAs in biomineralization. Compared with CAs of the other clades, the protein sequences of clade I are more conserved, with a sequence identity between species ranging between 52 and 82% (Supplementary Figure 1).

Clades II and III together form a monophyletic clade with high bootstrap and posterior probability support. Most of the complete CAs of clades II and III have an identifiable signal peptide (Supplementary Table 2). Two to six CAs per species are found in clade II, and the CAs of each subclass are monophyletic sister clades. Within the subclass clades, the CAs of the species are not monophyletic but intermixed. Intra-clade divergence in clade II is higher compared with the divergence of CAs in clade I (Supplementary Figure 2). Besides SciCA6, GcoCA6, and CspCA4, all proteins in this clade have a GPI anchor and therefore are predicted to be membrane-bound. The three zinc-binding histidine residues are generally conserved; only *SciCA4* is probably not catalytic due to a His-Asp replacement of the third catalytic histidine (Supplementary Table 2). Clade II includes two calcaronean CAs with a demonstrated expression in sclerocytes (SciCA2, LcoCA3, Voigt et al., 2014).

Clade III contains CAs from both subclasses, but not from all species; CAs from *S. ciliatum*, *G. compressa*, and *C. coriacea* are missing. These CAs possess a signal peptide but lack GPI anchors and therefore are secreted. The three zinc-binding residues are present in all CAs in this clade, so they are likely catalytic.

Clade IV forms a sister clade to a clade containing the *H. sapiens* CA X and XI and a CA from the urchin *Strongylocentrotu*s. In clade IV, each species possesses a single protein, which falls into two subclass-specific clades. Besides the previously described *SciCA9* and *LcoCA6* (Voigt et al., 2014), sequences of these clades’ CAs are partial. Where detectable, a signal peptide is present, and no GPI anchor was predicted, suggesting clade IV CAs are secreted (Supplementary Table 1). The three zinc-binding histidine positions are not conserved because the first histidine is substituted with arginine and the third histidine with glutamine in the sequences that cover this region of the protein. Therefore, the proteins of this clade likely lost their catalytic function and can be considered to represent CARPs.

## Discussion

The inclusion of additional CAs from both calcareous sponge subclasses revealed four CA clades with a specific subcellular localization: Clade I with sclerocyte-specific, mitochondrial, or cytosolic CAs, clade II with (mostly) membrane-bound CAs, including, at least for Calcaronea, sclerocyte-specific proteins, clade III with secreted CAs, not present in some species, and clade IV, a clade of secreted calcareous sponge CARPs.

The fact that each of these functional CA clades contains CAs of both subclasses suggests that the last common ancestor of calcareous sponges already possessed ancestral CA proteins belonging to each clade. The phylogeny agrees with the previously reported three clades of calcaronean CAs (Voigt et al., 2014), but now a clear subdivision of clades II and III is evident.

However, especially deeper nodes in the tree are only weakly supported by either bootstrap, posterior probabilities, or both, hampering understanding of the relationship of poriferan and coral CAs. The difficulties in obtaining robust phylogenies for animal CAs are known (Le Roy et al., 2014) and probably not surprising for such a single gene-family dataset, considering that even phylogenomic studies with thousands of genes produce conflicting relationships among animal phyla (King and Rokas, 2017). Nonetheless, the phylogeny of CAs again suggests that CAs were independently recruited for biomineralization in non-Bilateria. None of the CAs that have been suggested to be directly involved in biomineralization in the stony coral *Stylophora* (SpiCA1, SpiCA2: Moya et al., 2008; Bertucci et al., 2011) or in the coralline demosponge *Astrosclera* (Jackson et al., 2007) is particularly closely related to the sclerocyte-specific CAs we report in the clades I and II. We focus our further discussion on the four clades of calcareous sponge CAs, which show moderate to good support values.

Gene orthology is most evident in clades I and IV, in which only one CA per species was observed. Clade I CAs display a conserved role in biomineralization, showing sclerocyte-specific expression in Calcinea (CspCA1, Figures 3G–L) and Calcaronea (Voigt et al., 2014). It seems, therefore, that the involvement of mitochondrial CAs in biomineralization in calcareous sponges is an ancient feature dating back to the origin of this subclass’s key innovation, i.e., the formation of calcitic spicules. Whether the lack of an identifiable mitochondrial target sequence in one species (*Leucosolenia*) is due to limitations in the prediction of such motifs or a modification of the basic pattern in Calcaronea remains an open question. In this CA group, some variation in the temporal–spatial expression patterns between species appears to have evolved. In *Clathrina* sp., thickener cells in later spicule formation stages also express this CA (Figures 2J–L). In the calcaronean *S. ciliatum*, however, thickener cells in later spicule formation stages cease CA expression, in agreement with the observations that these cells deposit little or no calcite in this species (Woodland, 1905; Ledger and Jones, 1977). In contrast, our observations in *Clathrina* sp. implies that thickener cells continue depositing calcite on the actines. Such thickening activity may be specific for this species or possibly for Calcinea in general.

In clade II, multiple gene-duplications and losses occurred in both Calcaronea and Calcinea. However, the fact that CAs of both subclasses are sister groups suggests that the duplication/loss events detected in this clade postdated the split of the two subclasses. The secreted, membrane-bound CAs SciCA2 of *Sycon* and LcoCA3 of *Leucosolenia* are sclerocyte-specific and involved directly in biomineralization (Voigt et al., 2014). We cannot yet identify a calcinean CA with a sclerocyte-specific expression in this clade. Thus, possibly, the recruitment for biomineralization of secreted CAs happened only in the subclass Calcaronea. Alternatively, our ISH experiments, which were limited by material availability, failed to provide a clear signal, hindering the interpretation of the expression patterns in this subclass. Additional experiments, including also other species of Calcinea, are required to address this question.

Clade III (secreted CAs) lacks CAs of *Sycon*, *Grantia*, and *C. coriacea*. In the two latter species, of course, CAs of this clade may not have been expressed in the sampled specimens hampering their detection in the transcriptomes. However, in the genome of *S. ciliatum*, a CA of this clade is also missing, pointing to a loss of secreted CAs in some calcareous sponge species. In this context, it seems relevant that for both, *Sycon* and *Grantia*, one CA in clade II (SciCA6, GcoCA6, respectively) lacks a GPI anchor, that is typical for other CAs of this clade, so these two CAs seem to be secreted. Possibly, they could have functionally replaced the now missing secreted CAs of clade III.

Calcareous sponge CARPs (clade IV) are easily identifiable orthologous proteins (one gene per species) whose catalytic function was already lost in the common ancestor of calcareous sponges. We conclude this from the observation that two of the zinc-binding histidines were replaced with the same amino acids in all CAs in this clade, supporting the hypothesis of a single loss of the CA activity in these CAs. CARPs of other invertebrates also show the same amino acid replacement (Le Goff et al., 2016), although they are not phylogenetically closely related. The function of CARPs in calcareous sponges remains unknown. In the fully grown sponges studied here, CARPs had low expression levels compared with most other CAs in the same species, and the obtained sequences were incomplete (Supplementary Table 2). However, in *Sycon*, expression of the CARP *SciCA9* peaks during early post-settlement stages, suggesting a role of these calcareous sponge CARP proteins in early post-larval life stages (Voigt et al., 2014).

In stony corals, the best-studied non-bilaterian animals regarding their biomineralization, only a few CAs have a documented expression in the calcifying tissues. In *Stylophora*, for example, two carbonic anhydrases, SpiCA1 and SpiCA2 (Figure 2), are expressed by calcifying cells (Moya et al., 2008; Bertucci et al., 2011). Because both of these have a signal peptide (Del Prete et al., 2019) and SpiCA2 was also found in the coral skeletal matrix (Drake et al., 2013), these CAs seem to be secreted or membrane-bound forms. The cytosolic SpiCA3 is expressed ubiquitously in all tissues, not only in calcifying cells (Del Prete et al., 2019). Although the role of this intracellular CA in coral biomineralization remains uncertain (Del Prete et al., 2019), our results confirm that intracellular mitochondrial/cytosolic CAs are an essential component of the calcareous sponge’s biomineralization tool kit. Accordingly, mitochondrial or cytosolic carbonic anhydrases (clade I) were recruited for biomineralization in the last common ancestor of extant calcareous sponges. This suggests that metabolic carbon may be an important constituent of the calcareous sponge spicule’s carbonate. The expression pattern in later stages of spicule formation may be subclass-specific and may be correlated to the deposition of calcite by thickener cells on the growing spicules. Secreted, membrane-bound CAs involved in biomineralization only were identified in Calcaronea, but further studies are required to investigate their role in calcification in Calcinea. Future studies could investigate the detailed role of CAs in the biomineralization process of calcareous sponges, for example, by comparing the enzymatic activity of biomineralizing versus non-biomineralizing CAs and tracing the carbon source of the molecules that are transformed by these enzymes.

## Data Availability Statement

The data presented in this study are deposited in the European Nucleotide Archive (ENA), study accession PRJEB41034, and in the LMU Open data repository ( 10.5282/ubm/data.202).

## Author Contributions

OV conceived the study and drafted the manuscript. BF generated the data. OV, CG, and CK performed the ISH experiments. OV and SV analyzed the data. GW provided the resources. SV and GW revised the manuscript. All authors contributed to the article and approved the submitted version.

## Conflict of Interest

The authors declare that the research was conducted in the absence of any commercial or financial relationships that could be construed as a potential conflict of interest. The reviewer AR declared a past co-authorship with one of the authors GW to the handling editor.

## Footnotes

1 https://github.com/TransDecoder

2 https://www.geneious.com
