# Supplementary figures and images for: Carbonic Anhydrases: An Ancient Tool in Calcareous Sponge Biomineralization
Source: Front Genet. 2021 Apr 7;12:624533. doi: 10.3389/fgene.2021.624533 (PMC8058475; doi:10.3389/fgene.2021.624533)

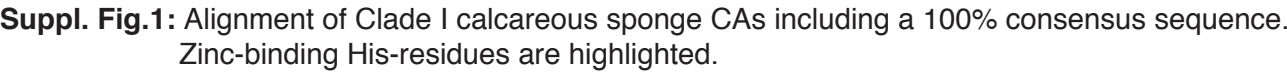

Supplement: Supplementary file 1 [file Data_Sheet_1.zip › Supplementary Figure 1.pdf]

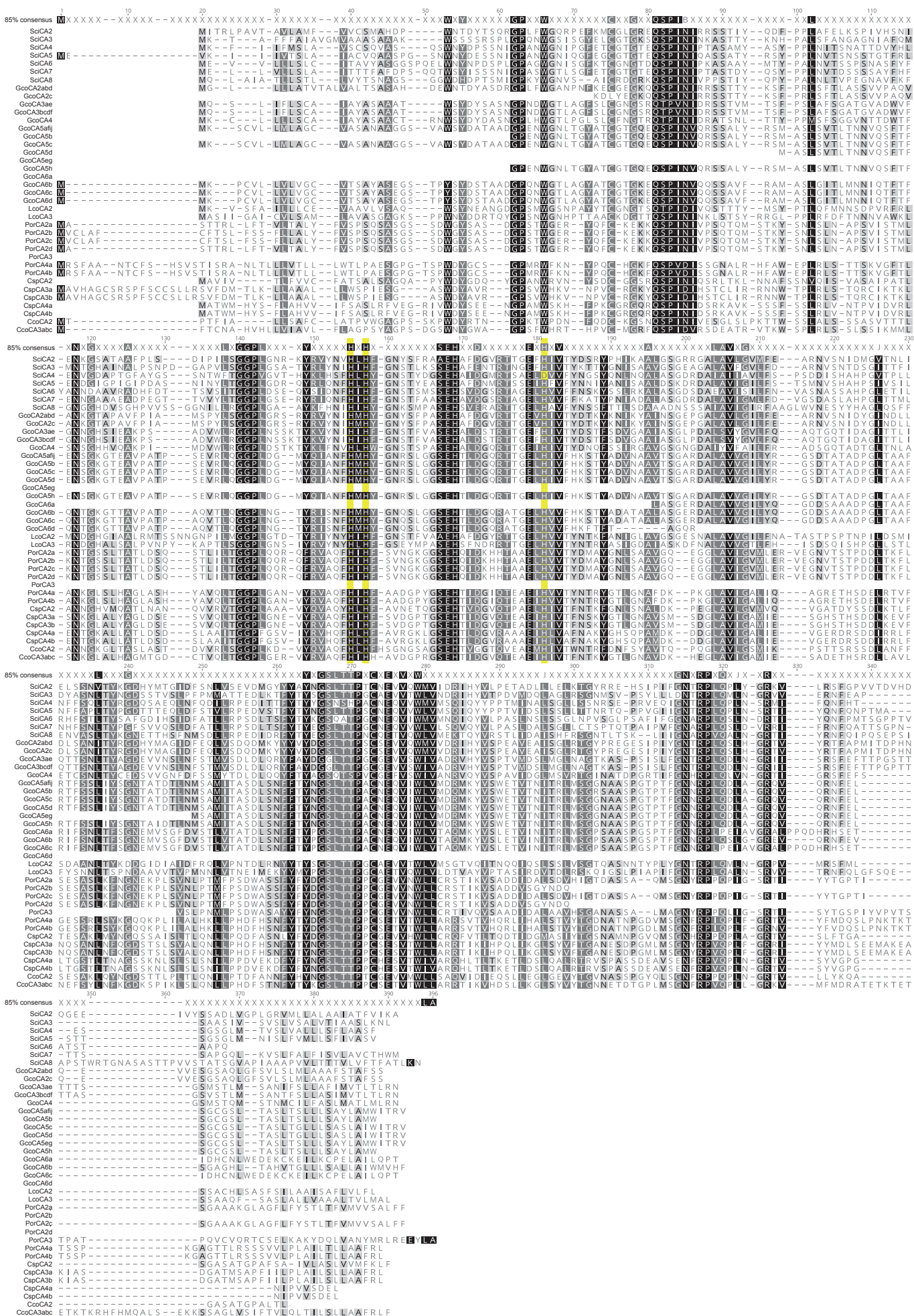

Supplement: Supplementary file 1 [file Data_Sheet_1.zip › Supplementary Figure 2.PDF]

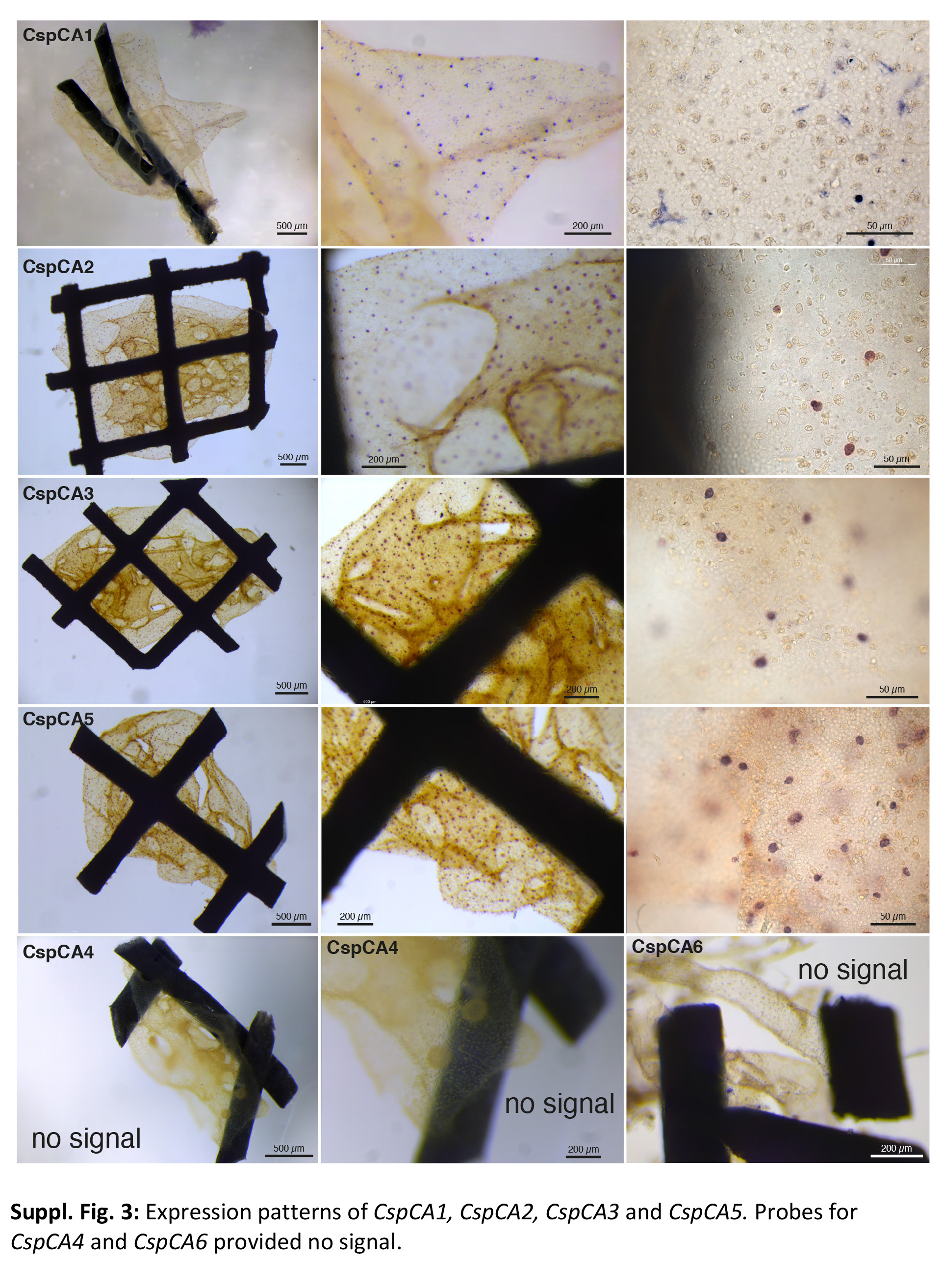

Supplement: Supplementary file 1 [file Data_Sheet_1.zip › Supplementary Figure 3.JPEG]
